# Interaction Between Cortical Auditory Processing and Vagal Regulation of Heart Rate in Language Tasks: A Randomized, Prospective, Observational, Analytical and Cross-Sectional Study

**DOI:** 10.1038/s41598-019-41014-6

**Published:** 2019-03-12

**Authors:** Viviane B. de Góes, Ana Claúdia F. Frizzo, Fernando R. Oliveira, David M. Garner, Rodrigo D. Raimundo, Vitor E. Valenti

**Affiliations:** 1 Autonomic Nervous System Center (CESNA), Department of Speech, Language and Hearing Therapy, UNESP, Marilia, Brazil; 2 Department of Maternal and Child Health, USP, Sao Paulo, Brazil; 3 Cardiorespiratory Research Group, Department of Biological and Medical Sciences, Faculty of Health and Life Sciences, Oxford Brookes University, Headington Campus, Oxford, OX3 0BP United Kingdom; 4 Laboratory of Design and Scientific Writing, School of Medicine of ABC, Santo Andre, SP Brazil

## Abstract

Cortical auditory evoked potentials (CAEP) throughout a language task is beneficial during psychophysiological evaluation to advance identification of language disorders. So as to better comprehend human communication and to provide additional elements for neuropsychological examinations we aimed to (1) examine the influence of language tasks on cortical auditory processing and vagal control of heart rate and (2) to verify a possible association between the parasympathetic cardiac regulation and cortical auditory processing in language tasks. This study was completed with 49 women. The subjects were separated into two groups: (1) phonological language tasks (N = 21) and (2) semantic (N = 21) language tasks. Heart rate variability (HRV) and CAEP were evaluated before and after the tests. HRV reduced (small effect size) and P3 wave latency increased after the phonological task. Identical variables were significantly correlated after the phonological task and linear regression indicated significant interaction between pNN50 (percentage of adjacent RR intervals with a difference of duration greater than 50 milliseconds) and P3 latency (16.9%). In conclusion, phonological language tasks slightly reduced parasympathetic control of HR and increased cognitive effort. The association between HRV and CAEP are anticipated to be involved in this mechanism.

## Introduction

Human verbal communication is correlated to the auditory system, which significantly supports the development of language skills. This relationship between auditory processing and verbal communication is vital for social interaction. The development of the autonomic nervous system delivers a neural platform for social behavior and affects human behavior^1,2^.

The autonomic nervous system gauges risk and defensive limbic structure interactions with social engagement and visceral states. Consequently, social communication can be expressed through social engagement modulated by the autonomic nervous system only when the defensive circuits of fight, flight or freeze behaviors are inhibited^2^. This control mechanism is similarly detected in auditory tests, when the individual must be actively engaged during the tasks for detecting target sounds^3,4^.

According to Lawrence and Barry^5,6^, fluctuations in heart rate (HR) are induced by auditory stimulus and HR accelerates with increased cognitive load. Since 1996, several studies have applied HR variability (HRV) as a well-recognized technique to non-invasively evaluate autonomic HR regulation^7^. Yet, it is unclear if HRV and cortical auditory processing respond to identical stimulus at comparable intensities.

In this way, auditory information processing is an essential parameter for social function examination. It is involved with cognitive domains such as executive function, memory and language. Cortical auditory processing permits the individual to understand language and contributes to their expression which can be evaluated through cortical auditory evoked potentials (CAEP)^3,4^. The executive language task is enforced in neuropsychological examinations, since it evaluates cognitive and language skills and assists in the neurological diagnosis of children, adults and the elderly. Language tasks involve the involvement of frontal and temporal cortical areas, which are appropriate for the cognitive domains mentioned directly above^8^.

Overall, executive language tasks may be divided into phonological and semantic tasks. In the phonological task, the patient is requested to reply with the highest number of words with a specific letter in an appropriate period (60 seconds). The most frequent responses are the letters F, A and S^9^. The phonological verbal fluency necessitates the use of non-habitual strategies, inhibiting the incorrect response, which demands greater cognitive effort. Numerous studies demonstrate that the phonological verbal fluency promotes greater activity of the frontal cortex^10^. The semantic task requests the patient to respond with the uppermost number of words with a specific semantic category in a suitable duration (again, 60 seconds). Animal is the most used semantic category^9^. Semantic verbal fluency permits the performance of semantic associations and meaning of words, dependent on memory and semantic knowledge. It has been revealed that during semantic tasks there is greater activity in the temporal cortex^11^.

So, we conjectured that the cortical auditory processing is connected to HRV and that executive language tasks adjust the performance of CAEP since it elicits greater cognitive effort, resulting in lower parasympathetic HR modulation. This study sought to answer these related queries: Does the cortical auditory processing and autonomic nervous system respond to the same stimulus at the same intensity? Can the executive language tasks change CAEP variables and HRV?

These two questions are necessary to further comprehend human communication and to provide the rudiments for neuropsychological examinations concerning the interaction between communication and autonomic function, allowing the development of therapeutic methods to improve language disorders.

Moreover, studying the relationship between CAEP and HRV conveys relevant mechanisms concerning clinical evaluation in subjects with neurological impairments with social function impairment. A possible association between autonomic nervous system and cortical auditory processing assists the clinicians to pursue further procedures or techniques to identify language impairment.

So, we proposed to (1) to evaluate the influence of language tasks on CAEP and vagal control of HR and (2) to scrutinize possible associations between the parasympathetic cardiac regulation and cortical auditory processing for language tasks.

## Results

Table 1 illustrates the values for baseline diastolic and systolic arterial pressure, age, height, mass, body mass index, hip, abdominal and waist circumferences, waist-to-hip ratio. All are within normal physiological standards^12,13^.

**Table 1:** Age, height, mass, body, body mass index, hip, abdominal and waist circumference, waist-hip ratio, systolic and diastolic arterial pressure of the subjects and normal physiological standards^12,13^.

Variables	Mean + standard deviation	Normal physiological standards
Age (years)	22.52 ± 2.82	—
Height (m)	1.62 ± 0.06	—
Mass (kg)	61.54 ± 10.72	—
BMI (kg/m^2^)	23.2 ± 3.69	18.5 e < 30
Waist circunference (cm)	74.14 ± 9.44	<80
Abdominal circumference (cm)	83.11 ± 8.39	<90
Hip circumference (cm)	99.9 ± 10.95	—
Waist-hip ratio	0.74 ± 0.06	≤0.85
SAP (mmHg)	107.61 ± 8.3	<120
DAP (mmHg)	68.09 ± 9.28	<80

Mean ± standard-deviation; BMI: body mass index; SAP: systolic arterial pressure; DAP: diastolic arterial pressure; m: meters; kg: kilograms; cm: centimeters; mmHg: millimeters of mercury.

**Figure 1:**
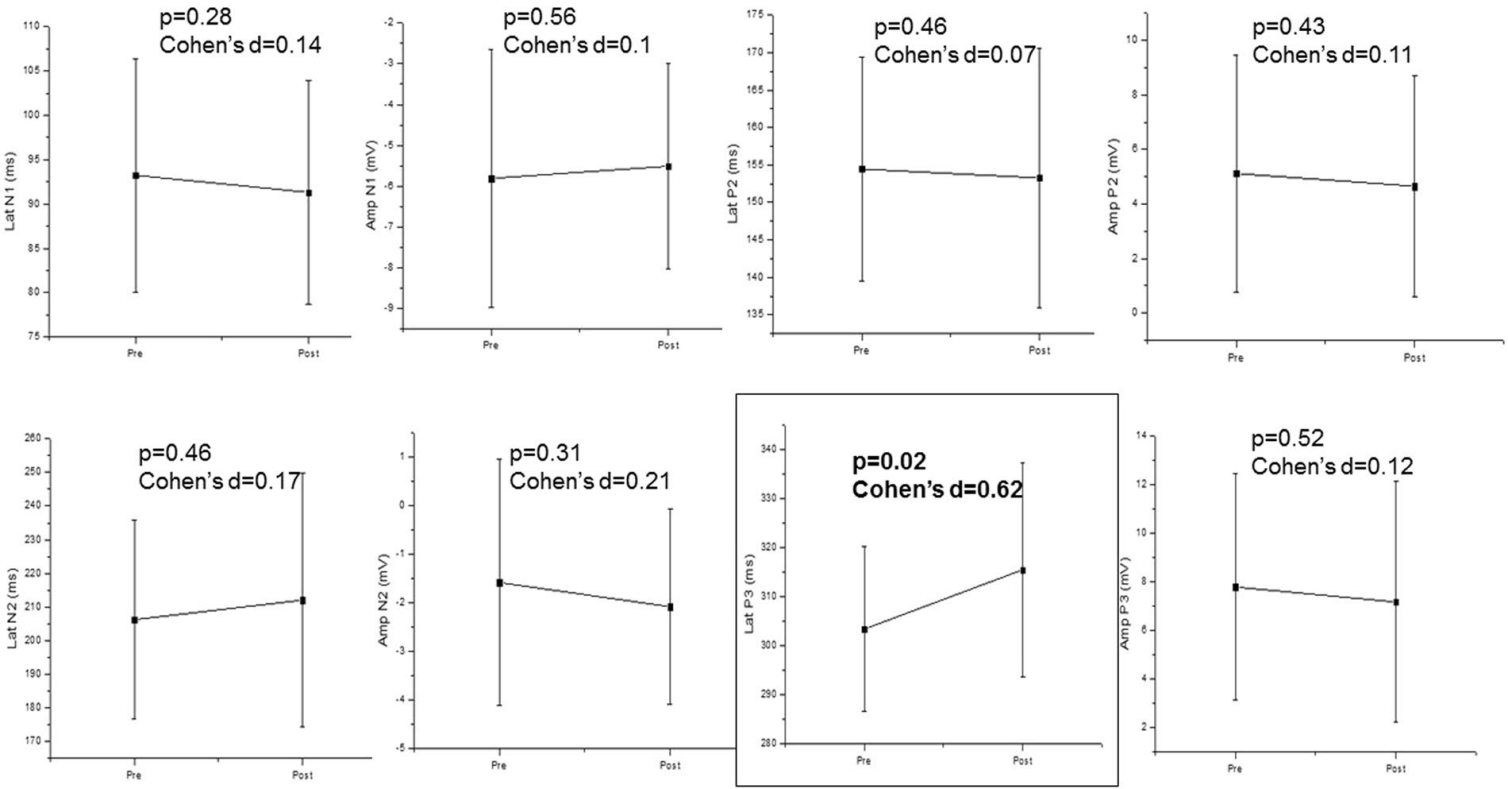
CAEP components before (pre) and after (post) the phonological task. Mean ± Standard deviation; Lat: latency; Amp: Amplitude; N1: N100; N2: N200; P2: P200 P3: P300.

When comparing CAEP components before and directly after the phonological tasks, we found a significant increase in latency of P3 wave (medium effect size), while no significant differences were achieved for N1, P2 and N2 amplitude and latencies (Fig. 1).

**Figure 2:**
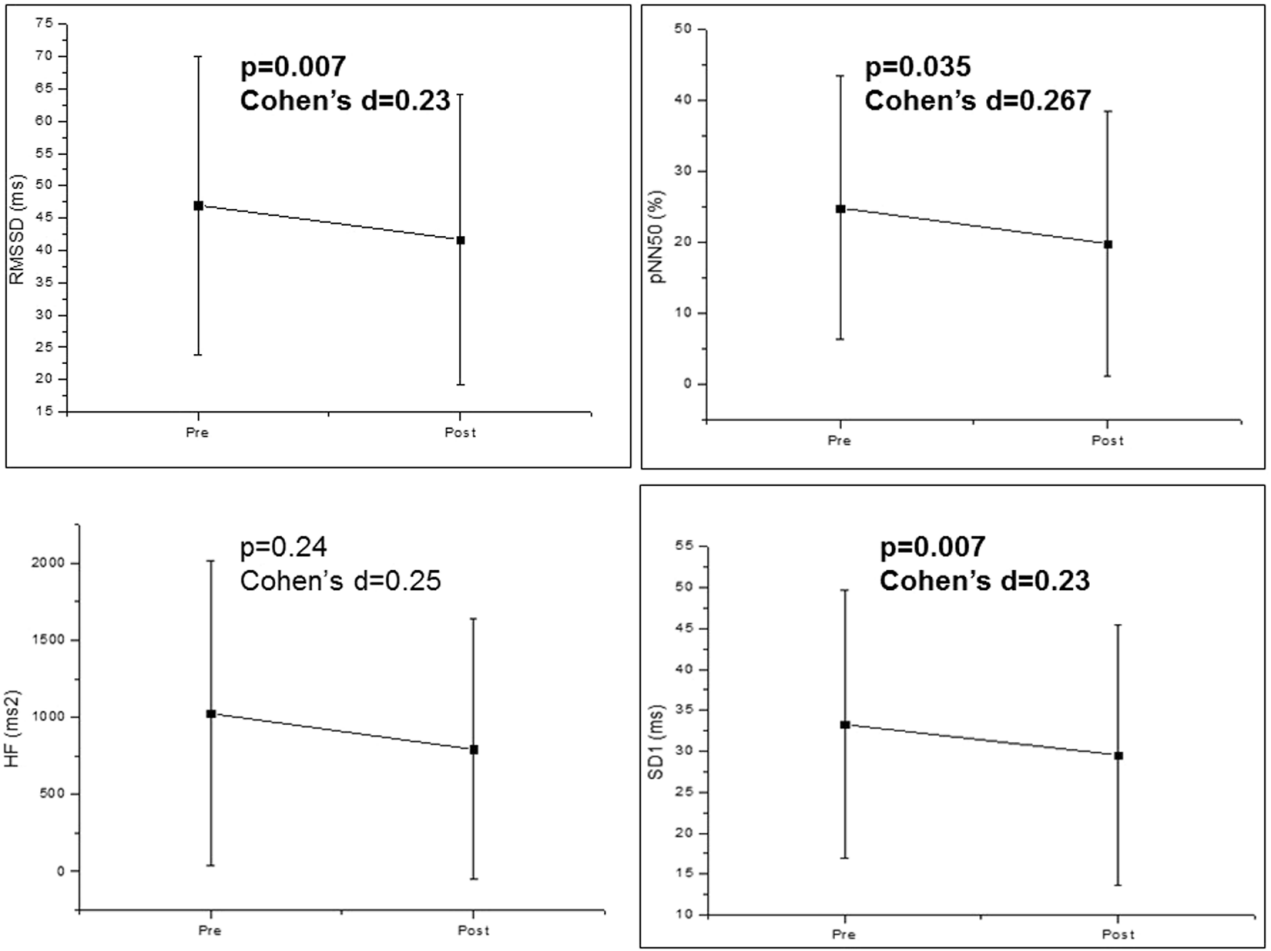
HRV indices before (pre) and after (post) the phonological task. Mean ± Standard deviation; RMSSD: root-mean square of differences between adjacent normal RR intervals in a time interval; pNN50: percentage of adjacent RR intervals with a difference of duration greater than 50 ms; HF: high frequency; ms: milliseconds; SD1: standard deviation of the instantaneous variability of the beat-to-beat heart rate.

In the identical tasks, we accomplished significant changes in HRV after the tasks. There were significant decreases of RMSSD, pNN50 and SD1 indices precisely after the phonological tasks. Yet, it only attained a small effect size (Fig. 2).

To evaluate the association between vagal HR control and cortical auditory processing in the phonological task we performed correlation analysis before and after the task. Table 2 reveals no significant correlation between HRV indices and CAEP components before the task.

**Table 2:** Correlation coefficient between CAEP and HRV before the phonological and semantic tasks.

Variable	Phonological		Semantic	
	r	P	r	p
**Lat N1**				
RMSSD	0.2744	0.228	0.1165	0.615
pNN50	0.2654	0.244	0.1801	0.180
HF (ms²)	0.1611	0.485	0.0384	0.868
SD1	0.2744	0.228	0.1165	0.615
**Amp N1**				
RMSSD	−0.1228	0.596	−0.2416	0.291
pNN50	−0.0799	0.730	−0.1878	0.414
HF (ms²)	−0.2982	0.189	−0.2208	0.336
SD1	−0.1228	0.596	−0.2416	0.291
**Lat P2**				
RMSSD	0.2830	0.213	0.3873	0.082
pNN50	0.2291	0.317	0.2985	0.188
HF (ms²)	0.2356	0.303	0.2606	0.254
SD1	0.2830	0.213	0.3873	0.082
**Amp P2**				
RMSSD	0.1662	0.471	0.3519	0.117
pNN50	0.1332	0.565	0.4062	0.067
HF (ms²)	0.0175	0.939	0.3273	0.147
SD1	0.1662	0.471	0.3519	0.117
**Lat N2**				
RMSSD	−0.0598	0.796	0.2976	0.190
pNN50	−0.1339	0.563	0.2234	0.330
HF (ms²)	−0.1423	0.538	0.2105	0.359
SD1	−0.0598	0.796	0.2976	0.190
**Amp N2**				
RMSSD	0.0896	0.699	0.0571	0.805
pNN50	0.1403	0.544	0.0838	0.717
HF (ms²)	0.0429	0.853	−0.0260	0.911
SD1	0.0896	0.699	0.0571	0.805
**Lat P3**				
RMSSD	−0.2263	0.323	0.2195	0.339
pNN50	−0.2895	0.203	0.2593	0.256
HF (ms²)	0.0403	0.862	0.1974	0.391
SD1	−0.2263	0.323	0.2195	0.339
**Amp P3**				
RMSSD	0.2130	0.353	−0.1455	0.529
pNN50	0.2306	0.314	−0.0890	0.701
HF (ms²)	0.3072	0.175	0.0156	0.946
SD1	0.2130	0.353	−0.1455	0.529

Amp: amplitude; Lat: latency; RMSSD: root-mean square of differences between adjacent normal RR intervals in a time interval; pNN50: percentage of adjacent RR intervals with a difference of duration greater than 50 ms; HF: high frequency; ms: milliseconds; SD1: standard deviation of the instantaneous variability of the beat-to-beat heart rate.

In contrast, we found a correlation between HRV and CAEP after the task. There was moderate correlation of P3 latency with RMSSD, pNN50 and SD1 indices after the phonological task. There was weak correlation of N1 latency with RMSSD, pNN50 and SD1 indices after the semantic task (Table 3).

**Table 3:** Correlation coefficient between CAEP and HRV after the phonological and semantic tasks.

Variable	Phonological		Semantic	
	R	p	R	p
**Lat N1**				
RMSSD	0.2134	0.353	**0.4660**	**0.033**
pNN50	0.1315	0.570	**0.4430**	**0.044**
HF (ms²)	−0.0029	0.989	0.3703	0.098
SD1	0.2134	0.353	**0.4660**	**0.033**
**Amp N1**				
RMSSD	−0.2208	0.336	−0.2935	0.196
pNN50	−0.1345	0.561	−0.3115	0.169
HF (ms²)	−0.0325	0.888	−0.2260	0.324
SD1	−0.2208	0.336	−0.2935	0.196
**Lat P2**				
RMSSD	−0.0058	0.979	0.1059	0.647
pNN50	−0.0312	0.893	0.1367	0.554
HF (ms²)	−0.0806	0.728	−0.0182	0.937
SD1	−0.0058	0.979	0.1059	0.647
**Amp P2**				
RMSSD	0.2403	0.294	−0.0636	0.784
pNN50	0.2014	0.381	0.7841	0.696
HF (ms²)	0.1306	0.572	−0.0429	0.853
SD1	0.2403	0.294	−0.0636	0.784
**Lat N2**				
RMSSD	−0.1086	0.639	0.2912	0.200
pNN50	−0.1191	0.607	0.3285	0.146
HF (ms²)	−0.0296	0.898	0.1371	0.553
SD1	−0.1086	0.639	0.2912	0.200
**Amp N2**				
RMSSD	0.1390	0.548	−0.1351	0.559
pNN50	0.1890	0.411	−0.1503	0.515
HF (ms²)	0.1208	0.601	−0.1598	0.489
SD1	0.1390	0.548	−0.1351	0.559
**Lat P3**				
RMSSD	**−0.6153**	**0.003**	0.3241	0.151
pNN50	**−0.5720**	**0.006**	0.3796	0.089
HF (ms²)	−0.4985	0.02	0.3001	0.186
SD1	**−0.6153**	**0.003**	0.3241	0.151
**Amp P3**				
RMSSD	0.3156	0.163	−0.1831	0.426
pNN50	0.3339	0.139	−0.1860	0.419
HF (ms²)	0.3436	0.127	−0.2299	0.316
SD1	0.3156	0.163	−0.1831	0.426

AMP: amplitude; LAT: latency; RMSSD: root-mean square of differences between adjacent normal RR intervals in a time interval; pNN50: percentage of adjacent RR intervals with a difference of duration greater than 50 ms; HF: high frequency; ms: milliseconds; SD1: standard deviation of the instantaneous variability of the beat-to-beat heart rate.

Table 4 demonstrates the simple linear regression analysis regarding HRV and CAEP after the phonological task. We established a significant interaction between pNN50 and P3 latency (16.9%).

**Table 4:** Linear regression between CAEP and HRV after the phonological task.

Models	β	95% C.I.	p	R^2^-adjusted
**1- LAT P3**				
**RMSSD**	−0.370	−0.769; 0.0289	0.067	−0.023
**2- LAT P3**				
**pNN50**	**−0.528**	**−1.018; −0.037**	**0.036**	**0.169**
**3- LAT P3**				
**SD1**	−0.523	−1.086; 0.0403	0.671	0.121

R-ADJUSTED: coefficient of determination of the percentage of variation; β: Beta; C.I: confidence interval; LAT: latency; RMSSD: root-mean square of differences between adjacent normal RR intervals in a time interval; pNN50: percentage of adjacent RR intervals with a difference of duration greater than 50 ms; HF: high frequency; ms: milliseconds; SD1: standard deviation of the instantaneous variability of the beat-to-beat heart rate.

**Figure 3:**
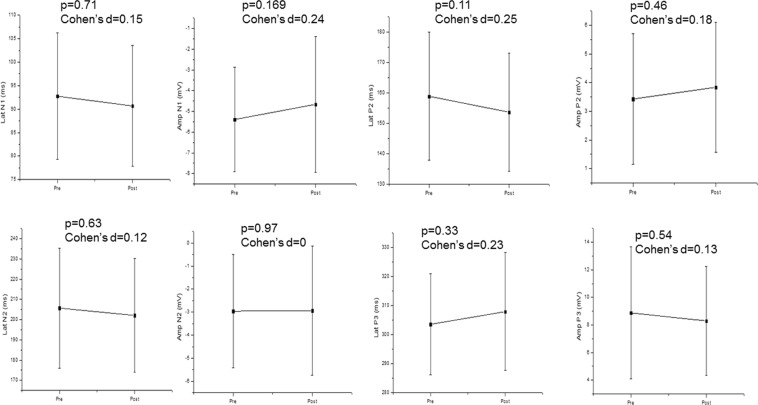
CAEP components before (pre) and after (post) the semantic task. Mean ± Standard deviation; Lat: latency; Amp: Amplitude; N1: N100; N2: N200; P2: P200 P3: P300.

**Figure 4:**
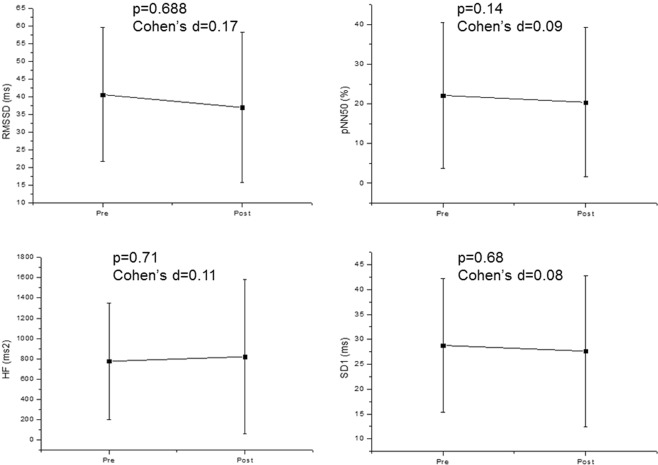
HRV indices before (pre) and after (post) the semantic task. Mean ± Standard deviation; RMSSD: root-mean square of differences between adjacent normal RR intervals in a time interval; pNN50: percentage of adjacent RR intervals with a difference of duration greater than 50 ms; HF: high frequency; ms: milliseconds; SD1: standard deviation of the instantaneous variability of the beat-to-beat heart rate.

Regarding the semantic task, we revealed no significant association between HRV and CAEP. According to Fig. 3 there was no significant change in CAEP components before and after the task. Figure 4 confirmed that HRV did not change significantly after the semantic task compared to baseline values before the task.

In regard to the association between HRV and CAEP in the semantic tasks, there were no significant correlations (Tables 2 and 3).

**Figure 5:**
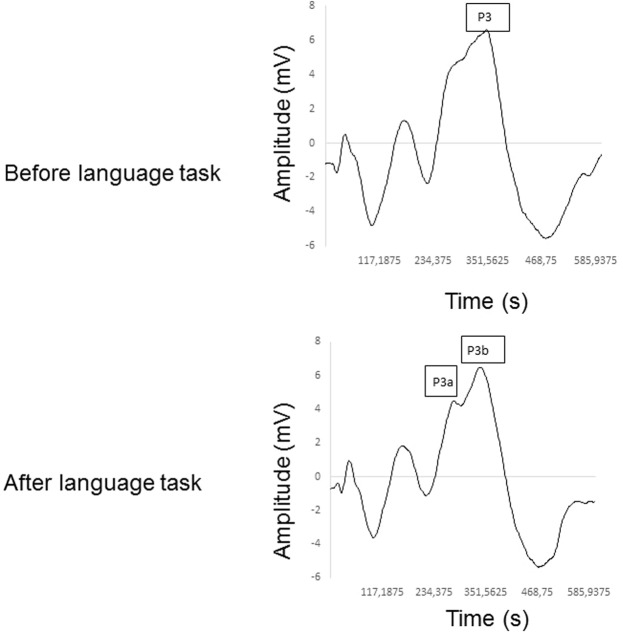
Qualitative analysis of the CAEP components before (pre) and after (post) the phonological task, showing the double peak in P3 with its subcomponents (P3a and P3b). Mean ± Standard deviation; Lat: latency; Amp: Amplitude; P3: P300.

Figure 5 determines the mean of the CAEP components before and after the phonological language task. It is important to highlight the double peak formation of the P3 wave after the phonological and semantic tasks in 18 subjects, revealing the P3a and P3b subcomponents.

Correction for multiple comparisons in correlation tests through Bonferroni post-hoc achieved p < 0.001 for significance.

## Discussion

Our study was instigated to consider the interaction between HR autonomic regulation and cortical auditory processing in language tasks. As foremost accomplishments, we report that: (1) language tasks induced cortical auditory responses and slight autonomic deviations; (2) the association between CAEP and HRV helps to explain this mechanism; (3) the phonological task offered more influence on this interaction compared to the semantic task as a result of more intense cognitive effort; (4) there was moderate interaction between exogenous/sensorial CAEP components and HRV during the semantic task.

Still, auditory processing and HR autonomic modulation involve particular areas of the nervous system. To provide further evidence concerning this association, previous studies present a considerable association between the autonomic and central nervous systems^14–16^.

Nakamura *et al*.^16^ considered the interaction between auditory processing and autonomic function in animals. These investigators originated an electrophysiological experiment during introduction to classical music (“Träumerei” from Kinderszenen Op.15-7, R. Schumann). The gastric vagal nerve activity increased during auditory stimulation, which was accompanied by activation of c-Fos protein expression in the auditory cortex; subsequently, indicating an association between cortical auditory processing and the parasympathetic nervous system. Latterly, Martiniano *et al*.^15^ verified intensification of the autonomic responses to anti-hypertensive medication elicited by musical auditory stimulus, signifying that increased gastric vagal activity induced by auditory stimulus enhanced pharmacological absorption in the gastrointestinal system.

Likewise, Marcomini *et al*.^14^, analyzed the association between resting HRV and CAEP in male subjects. This study conveyed significant association of the parasympathetic control of HR with the CAEP waves related to external stimulus. So, we speculated that the autonomic control of heart rhythm interacts with cortical sound processing in diverse situations.

Nonetheless, it was unclear if the association between the autonomic nervous system and cortical auditory processing were involved in language tasks, which are part of the neuropsychological assessments^17^. Additional information concerning this physiological relationship is pertinent for improvement of language disorder treatments. Our results reveal that the phonological language task induced autonomic and cortical auditory changes due to more intense cognitive efforts, ensuing in decreased parasympathetic modulation. Equally, no significant change in HRV and CAEP were achieved in the semantic language task.

Consistent with our conclusions, following the phonological task, HRV slightly reduced and the latency of P3 wave increased. This response indicates that the stress during the phonological task is involved in this process, since reduced parasympathetic control of HR is related to stressful or traumatic processes^18,19^. Moreover, linear regression indicated that if the pNN50 index declined by 1%, the P3 latency would increase by 0.5 milliseconds.

The connection between HRV and CAEP detected after the phonological language task revealed that during this task the auditory perception represented by the P3 wave was mobilized by the parasympathetic modulation of HR, hence a submissive response. These results support the outcomes published by Lawrence and Barry^5,6^, which demonstrated changes in HR during auditory stimulus and the acceleration of HR according to the load of cognitive processing considered by the cortical auditory processing.

In view of the autonomic and cortical responses induced by the phonological task demonstrated in our investigation, we propose amalgamation of cortical areas processing, HR regulation and attention. Language tasks require the activity of non-habitual strategies, demanding greater cognitive effort^20^ and prefrontal cortex activation^21^, which is one of the principle regions related to the cognitive P3 component^22^. Activation of the prefrontal cortex encourages increases in sympathetic activity^23^. As a further significance, we advocate this physiological relationship to elucidate parasympathetic reduction and P3 latency increases during accomplishment of a cognitive effort task.

Also, we stated a double peak of the P3 wave after both language tasks in the majority of the subjects. The temporal cortex is significantly activated during semantic task^24,25^. The appearance of the P3a subcomponent reflects an increase in the pre-frontal cortex activity elicited by the cognitive task. Then, an increase in alertness and involuntary attention generates the P3b subcomponent. This parameter matches the volunteer’s active participation during target sound discrimination^3,4,26^. Though, the subcomponents are not present in all subjects, and as a consequence, we could not provide consistent descriptive statistics of P3a and P3b subcomponents.

Our data reinforced another study published by Resstel and Correa^27^. These authors presented a review that provided results from their laboratory indicating the effects of medial prefrontal cortex activation on cardiac autonomic modulation in rats. Amongst their main accomplishments, they highlighted the role of glutamate, acetylcholine and noradrenaline in the prefrontal cortex during baroreflex parasympathetic activation and stress-related cardiac responses inducing tachycardia. Yet, those physiological mechanisms are inconclusive for human subjects. So, our study uncovered an important individuality.

We revealed weak but significant association between vagal regulation of HR and cortical auditory processing (N1 wave) when the subjects performed the semantic task. The semantic task permitted the comprehension of semantic associations and meaning of words, making it easier to complete compared to the phonological task as it required a minor cognitive effort to retrieve the names of the animals^20,28–31^.

The exogenous/sensorial CAEP components are represented by P1, N1 and N2 waves. They reflect the acoustic and temporal characteristics of the stimulus and are necessary for auditory ranges assessment during auditory examination. Those components convey information regarding the arrival of auditory stimulus to the cortex, onset of cortical processing, it also indicates if the auditory signal was appropriately received in the auditory cortex^32^.

The weak association between exogenous/sensorial CAEP components (N1) during the semantic task and the presence of significant moderate interaction between P3 wave and HRV during phonological task suggest more intense cognitive effort during the phonological task compared to the semantic task.

Some findings in our investigation require highlighting. (1) HRV was recorded concurrently with CAEP to detect autonomic and cortical changes immediately after the language task and to authenticate the synchronization between the two systems. (2) We investigated only women to circumvent the effects of sexual hormones. We should be cautious with different genders when interpreting data. (3) We identified a small effect size for HRV changes induced by the phonological language task. So, we concluded that this specific task causes *slightly* significant autonomic changes. (4) We demonstrated significant correlation of all HRV indices with the P3 wave latency in the phonological task, supporting the involvement of the cognitive system. (5) Bonferroni correction for multiple comparison increased Type I error (p < 0.001). The values of “p” achieved in our results (between 0.003 and 0.006) are close to the value presented in the Bonferroni correction. It is worth highlighting that applying the Bonferroni correction may expand the Type II error. In this way, we considered the Spearman coefficient Rho between 0.5 and 1, which is sufficient to define an acute physiological response^33^.

Nevertheless, previous studies have revealed a correlation between HRV indices and CAEP components. When applying the Bonferroni correction, the “p” value to be considered is very low (p ≈ 0.001) thus confirming an acute response of the low intensity would be impractical. In addition, the significant values of “p” found here are close to the value presented in the Bonferroni correction, so if we apply the Bonferroni correction, we could expand the Type II error. An additional factor that we considered central together with the statistical significance was the clinical relevance. Biological data, especially human data, have revealed very high correlations and may be considered too good to be true. So, we considered the Spearman coefficient Rho = 0.6 sufficient to define an acute response to intervention in the phonological group.

This study delivers important mechanisms for neurological examination in patients with language disorders. They expose the association between social interaction and the autonomic nervous system^2,34,35^. Thus, explaining the categorization regarding the interaction between HR autonomic regulation and cortical auditory processing during a phonological language task. It is helpful in psychophysiological evaluations to advance clinical identification of language disorders.

Moreover, the association between cortical auditory processing and autonomic nervous system may support the multidisciplinary clinician to pinpoint a technique to detect language impairment. In summary, our data provides important elements regarding screening for language disorders, since autonomic evaluation during language tasks offers further physiological responses.

## Conclusion

A phonological language task slightly decreased vagal control of HR and increased latency of P3 wave. We advocate that the interaction between parasympathetic regulation of HR and cortical auditory processing are involved in this physiological response.

## Method

### STROBE Guidelines

Our study conforms to the STROBE (STrengthening the Reporting of OBservational studies in Epidemiology) guidelines. Our investigation contains details of the study design, setting, participants, variables, data sources, measurement, description of potential sources of bias, quantitative variables description, and statistical methods.

### Population study and Eligibility Criteria

**Figure 6:**
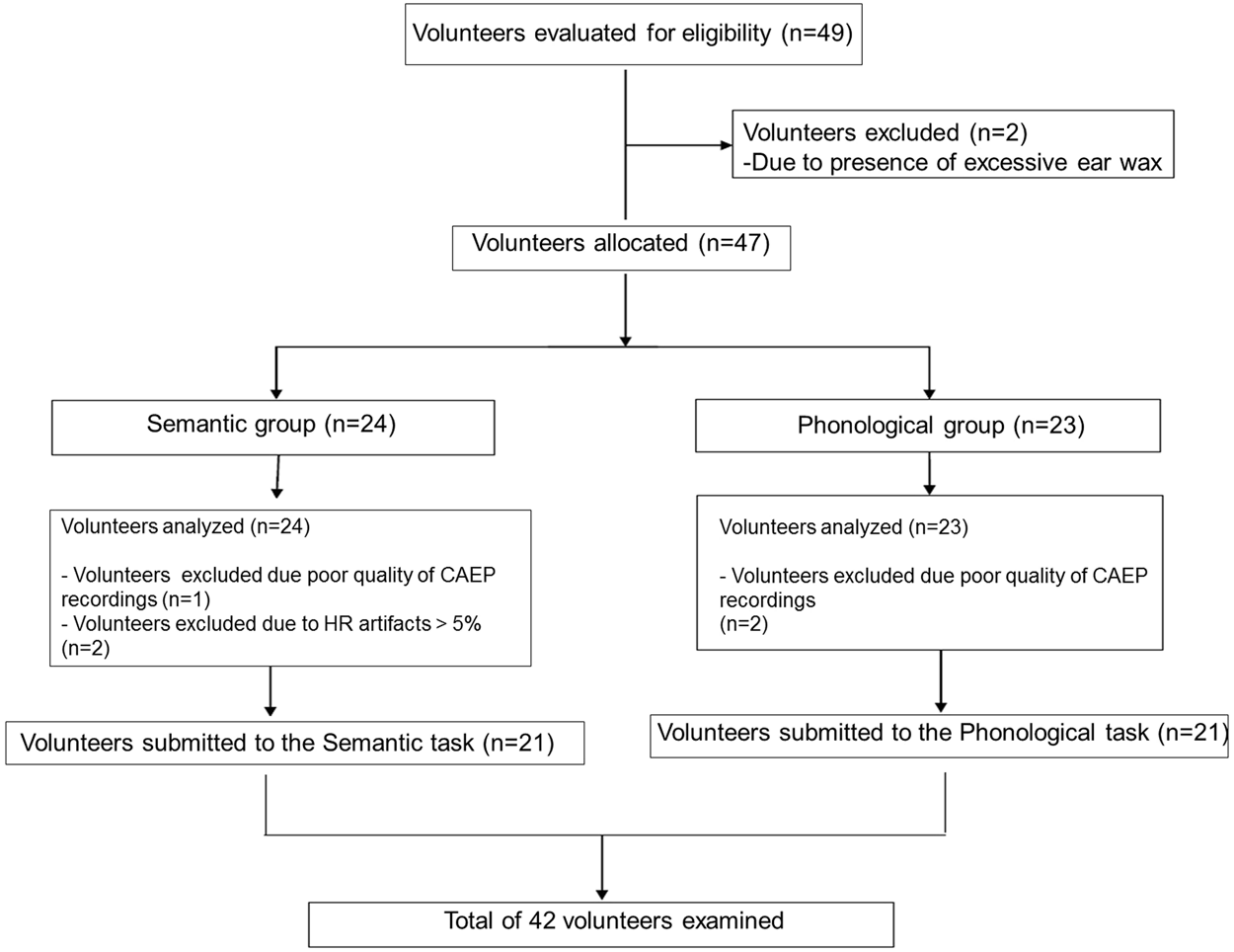
Flowchart.

This study was completed by 49 women. Seven subjects were excluded because they had excessive ear wax and presented artifacts during RR intervals recording and poor quality of CAEP recordings (Fig. 6). The remaining participants were randomly split based on language tasks (semantic or phonological). Before the experimental protocol, the subject selected a card that indicated semantic or phonological group. The phonological group (PG) was assigned 21 subjects who performed the phonological task while the semantic group (SG) was equally allotted 21 subjects that completed the semantic task.

We excluded subjects that conveyed cardiopulmonary, neurological, psychological related diseases, other related disorders that prohibited the participant performing experimental protocols and pharmacological treatments that influenced cardiac autonomic regulation and CAEP. We excluded women between the 10^th^ and 15^th^ days and between the 20^th^ and 25^th^ days of the menstrual cycle to eliminate influence of the luteal and follicular phase, respectively^36^.

### Ethical approval and informed consent

All experimental protocols were approved by the Research Ethics Committee in Research of UNESP/Marilia (Number 1.804.034) and a statement from the Committee explicitly stated that the methods were undertaken in accordance with the 466/2012 resolution of the National Health Council of December 12^nd^ 2012. Informed consent was attained from all participants.

### Study design and setting

This is a prospective, observational, analytical and cross-sectional study performed at the Faculty of Philosophy and Science at UNESP, Marilia, SP, Brazil.

### Bias

We completed all protocols under the same environments so as to address potential sources of bias. The data was recorded at an identical time of the day (between 13:00 and 17:00) to standardize circadian influences, in a room with temperature between 21 °C and 25 °C and humidity amid 40% and 60%. The subjects were instructed to avoid drinking coffee and ingesting other autonomic stimulants for 24 hours before the data collection and to maintain an empty bladder during the investigation.

The descriptive profile of the subjects was defined to characterize the sample, reduce the unpredictability of the variables, improving reproducibility and physiological interpretation. Before the start of the experimental procedures, subjects were documented according to age, mass (kg), height (m), systolic (mmHg) and diastolic arterial pressure (mmHg), waist (cm), abdominal (cm) and hip (cm) circumferences, waist-to-hip ratio and body mass index (BMI).

### Initial assessment and experimental protocols

Primarily, the HR monitor was placed on the volunteer’s chest. Their skin was cleaned with abrasive paste in the positioning region of the electrodes to perform the CAEP recordings. These were then secured with microporous tape, using electrolytic paste for improved electrical conductivity. Next, the participants were instructed to remain seated under spontaneous breathing in silence in a comfortable armchair for 10 minutes, avoiding conversation during this period.

**Figure 7:**
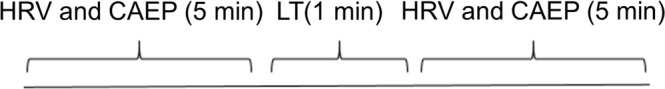
Experimental protocol. CAEP: cortical auditory evoked potential; HRV: heart rate variability; LT: language task.

After that, the experimental procedures were performed as follows (Fig. 7):

1. Pre-test – RR intervals and CAEP were recorded concurrently before the language task for five minutes under spontaneous breathing in silence;
2. Test – For the phonological task, the volunteers received the following instructions: “You will receive a letter and you will tell me the maximum number of words in Brazilian Portuguese that exist and begin with that letter in 60 seconds. You should avoid repeating words and using the augmentative and diminutive forms. The letter to be used is the letter A”.
  In the semantic task, the volunteers received the following instructions: “Tell me as many animals in Brazilian Portuguese as you can in 60 seconds. It can be aquatic, terrestrial and bird animals. You should avoid repeating them and using augmentative and diminutive forms”.
3. Post-test – Straight after, the task’s RR intervals and CAEP were recorded simultaneously for five minutes under spontaneous breathing in silence.

### Variables, data sources and outcome measures

#### HRV analysis

The portable RS800CX heart rate (HR) monitor was essential to record RR intervals with a sampling rate of 1 kHz. The RR intervals were transferred to the Polar Precision Performance program (v.3.0, Polar Electro, Finland). The Polar transmitter distinguishes all heart beats in the left ventricular muscle and the data recorded, transmitting the signal to the computer via an infrared method through a wireless technology. The software enabled the visualization and the extraction of a cardiac period (RR interval) file in “txt” format.

Details of HRV analysis have been published previously^37,38^ and follow directives from the Task Force^7^. Throughout RR interval recording, we monitored the respiratory rate that wavered between 9 and 13 cycles per minute.

To evaluate the parasympathetic regulation of HR we analysed pNN50 (percentage of adjacent RR intervals with a difference of duration greater than 50 milliseconds), RMSSD (root-mean square of differences between adjacent normal RR intervals) in the time domain, high frequency band of spectral analysis (HF: 0.15 Hz to 0.4 Hz) in absolute units in the frequency domain and the SD1 Poincaré plot (standard deviation of the instantaneous variability of the beat-to-beat heart rate). We employed the Kubios^®^ HRV v. 2.0 software to compute these indices^39^.

#### Audiological evaluation

We inaugurated audiological inspections and pure tone audiometry to exclude subjects with auditory disorders. The following assessments were performed in an identical soundproofed room. Initially, an auditory examination to obtain material on the patients’ medical history. Then, a pure tone audiometry to assess hearing thresholds (air and bone conduction). This examination was accomplished with a two-channel audiometer, Grason-Stadler (GSI) 61, with TDH-39. We omitted subjects with hearing impairments (tonal thresholds below 25 dBNA)^40^ in both ears and included a Type A tympanometric curve to characterize the normality of the tympanic bone system^41^.

#### Examination of cortical auditory evoked potential (CAEP)

The key purpose of this electrophysiological examination was to evaluate the integrity of the auditory pathway in the brain. Examination of CAEP was performed in a soundless room with the subject seated and instructed to remain alert. The oddball paradigm was enforced during electrophysiological recordings.

Electrophysiological assessment was initiated using the long-latency auditory evoked potential (P300a) recordings. Bio-logic Systems Corporation equipment was essential for the P300a recording. The active electrodes were located on the forehead (Fpz = ground electrode), the cranial vertex (Cz = active electrode), and the earlobes (reference electrode: A1 = LE and A2 = RE), according to the International 10–20 System, and headphones were suitably placed (TDH-39).

We examined the latency and amplitude of the P300 (P3). We enforced the protocols as previously published^42^.

### Study size

The sample size was computed through the online software provided by the website www.lee.dante.br, considering the RMSSD index as a variable. The significant difference in magnitude assumed was 14.11 milliseconds, with a standard deviation of 12.8 milliseconds, per alpha risk of 5% and beta of 80%. As the final significance, the sample size determined a minimum of 13 volunteers per group. We computed the sample size and it established a power of 83%.

### Statistical analysis

We completed a Shapiro-Wilk normality test to estimate the distributions. To equate HRV and CAEP before and after language tasks we applied the paired Student t-test or Wilcoxon test.

To compute the magnitude of differences between pre-task and post-task we calculated the effect size using Cohen’s *d*. Large effect sized was considered for values > 0.9, medium for values between 0.9 and 0.5 and small for values beneath 0.5^43^.

With the intention of evaluating the correlation between HRV indices and CAEP components we enforced the Spearman correlation coefficient. Strong correlations were considered for r greater than 0.75 and moderate correlations were considered for r between 0.5 and 0.75.

To study the effect of independent variables on dependent variables a simple linear regression model was constructed. The choice of the independent variables was achieved primarily by the correlation analysis, considering only the variables with a significant correlation (p < 0.05). A simple linear regression model was required to model the HRV indices as outcome variables. Predictors included continuous variables representing CAEP. The R^2^ was assessed to verify the coefficient of determination of the percentage of variation explained by the model. It diverges between 0 and 1 and indicates how much the model can elucidate the observed values. The higher the R², the more clarifying the model is, and the better it adjusts to the sample^44^. Statistical significance was considered at the level p < 0.05.
